# Prognostic Value of Serum Neutrophil Gelatinase-Associated Lipocalin in Acute Heart Failure: A Meta-Analysis

**DOI:** 10.31083/j.rcm2512428

**Published:** 2024-11-29

**Authors:** Zhendong Cheng, Xiaoxin Lin, Chaoxiang Xu, Zhilong Zhang, Naping Lin, Kefeng Cai

**Affiliations:** ^1^Department of Cardiology, The Second Affiliated Hospital of Fujian Medical University, 362000 Quanzhou, Fujian, China

**Keywords:** neutrophil gelatinase-associated lipocalin, acute heart failure, all-cause death, heart failure readmission, meta-analysis

## Abstract

**Background:**

Neutrophil gelatinase-associated lipocalin (NGAL) is not only a sensitive marker of acute kidney injury but may also be a prognostic marker of acute heart failure (AHF). This study aimed to investigate the relationship between serum NGAL and all-cause death (ACD) and the composite outcome of ACD or AHF readmissions in patients with AHF.

**Methods:**

The Embase, Cochrane Library, and PubMed databases were searched for articles focusing on serum NGAL and ACD and the composite outcome of ACD or AHF readmissions in patients with AHF. The hazard ratios (HRs) were pooled with random-effects models.

**Results:**

The results from 2428 patients from seven studies were pooled in this article. Higher NGAL was relevant to an increased risk of ACD (HR, 1.89; 95% CI, 1.38 to 2.61) and the composite outcome of ACD or AHF readmissions (HR, 2.92; 95% CI, 1.62 to 5.27) in patients with AHF.

**Conclusions:**

Serum NGAL has prognostic value for ACD and the composite outcome of ACD or AHF readmissions in AHF.

**The PROSPERO registration:**

CRD42022322057, https://www.crd.york.ac.uk/prospero/display_record.php?ID=CRD42022322057.

## 1. Introduction

Acute heart failure (AHF) is the most common cause of hospitalization in older patients (aged >65 years) and is associated with high mortality and readmission rates. The in-hospital mortality rate ranges from 4% to 10% [1, 2, 3, 4]. One-year mortality can be 25–30%, and the mortality or readmission rate can be more than 45% [2, 3, 5, 6, 7]. Therefore, AHF remains a life-threatening disease worldwide. Early risk stratification and patient classification are urgently needed to guide precise therapeutic and preventive strategies for AHF.

Using biomarkers, we can identify the risk level of patients with AHF. The gold standard biomarker for AHF is natriuretic peptides. Several powerful biomarkers have also emerged. Suppression of tumorigenicity 2, cardiac troponin, and galectin-3 have strong independent prognostic properties [8, 9, 10], whereas the application of others, such as neutrophil gelatinase-associated lipocalin (NGAL), copeptin, and cystatin C still requires further validation.

In AHF, acute kidney injury (AKI) is a very common complication that has a significant impact on prognosis [11, 12, 13, 14]. An acute-phase protein, NGAL, has emerged as an important biomarker of AKI [15, 16]. NGAL is not only a sensitive marker of AKI but also a prognostic marker of AHF [13, 14, 17, 18, 19, 20]. However, the prognostic value of NGAL in patients with AHF is inconsistent [21]. Owing to the limited number of clinical studies and no meta-analyses, we conducted a meta-analysis of the prognostic role of serum NGAL levels in patients with AHF.

## 2. Methods

This study was performed in accordance with PRISMA’s Preferred Reporting Items and was registered in the PROSPERO database (CRD42022322057).

### 2.1 Search Strategy

Meta-analysis of Observational Studies in Epidemiology Group protocol was followed in our study [22]. A literature search of the PubMed, Cochrane Library, and Embase databases was conducted up to the 28th of March, 2022. Using the Boolean operator “and”, we combined two search themes. The first theme was heart failure, including exploded versions of the medical subject headings heart failure, congestive heart failure, myocardial failure, and cardiac failure, heart decompensation. The second theme included lipocalin 2, lipocalin 2 protein, ngal protein, neutrophil gelatinase-associated lipocalin, lipocalin-2 protein, and neutrophil gelatinase-associated lipocalin. The Cochrane Library and Embase databases were searched using the same search string as PubMed.

### 2.2 Literature Inclusion and Exclusion Criteria

This analysis included studies that met the following criteria: (1) enrollment of patients with AHF (either *de novo* AHF or worsening congestive heart failure requiring hospitalization); (2) follow-up studies with adults (aged 18 or older); (3) an NGAL measurement was conducted in the serum; (4) the relationship between NGAL and all-cause death (ACD) was analyzed, possibly also for the composite outcome of ACD or AHF readmissions; (5) multivariable-adjusted hazard ratios (HRs) and 95% confidence intervals (CIs) are presented; (6) the language is English. Exclusion criteria: (1) research on patients with end-stage HF; (2) associated outcomes were provided only with unadjusted risks; (3) duplicated analyses or data.

### 2.3 Data Extraction and Quality Assessment

The candidate studies were evaluated and screened by two independent authors (XL and ZZ) based on predefined criteria. In the event of a disagreement, the two authors communicated with a third author (ZC) to reach a consensus.

The following basic information about the participants was recorded from each study: first author, country, mean age, follow-up period, year of publication, percentage of males, sample size, left ventricular ejection fraction (LVEF), serum NGAL, and estimated glomerular filtration rate (eGFR). Each included study was evaluated by two independent authors (KC and NL) according to the Newcastle–Ottawa Scale (NOS) [23]. The NOS assesses studies based on nine issues, meaning a total of nine iconic questions were evaluated as “Yes” (clear fit), “No” (not meeting the requests), and “Unclear”. Based on the evaluation issues, biases were classified into high risk, low risk, and unclear.

### 2.4 Statistical Analysis

The primary endpoint was serum NGAL-associated ACD risk, while the secondary endpoint was the composite outcome risk of ACD or AHF readmissions. A multivariable-adjusted HR and 95% CI for ACD and the composite outcome of ACD or AHF readmissions were collected from each study [24]. Cochran’s Q test and Higgins I-squared statistics were used to estimate the heterogeneity of the included studies. A *p*-value < 0.10 or *I*^2^ >50% indicated the existence of significant heterogeneity.

Owing to the high heterogeneity, we calculated the pooled HRs and 95% CIs using random-effects models. Using meta-regression analysis, we explored the potential influence of population characteristics on the primary endpoint. The heterogeneity in different studies was also examined using a sensitivity analysis. We did not perform subgroup analyses for ACD because available studies were limited. Egger’s test and Begg’s funnel plot were used to assess publication bias for ACD [25].

Statistical analyses were performed using STATA (StataCorp LP, College Station, TX, USA) version 12.0. RevMan (StataCorp LP, College Station, TX, USA), while 5.2 displayed the NOS assessment. Two-tailed *p*-values were calculated, and 0.05 was designated as the level of statistical significance.

## 3. Results

### 3.1 Literature Search Results and Characteristics

**Fig. 1.**
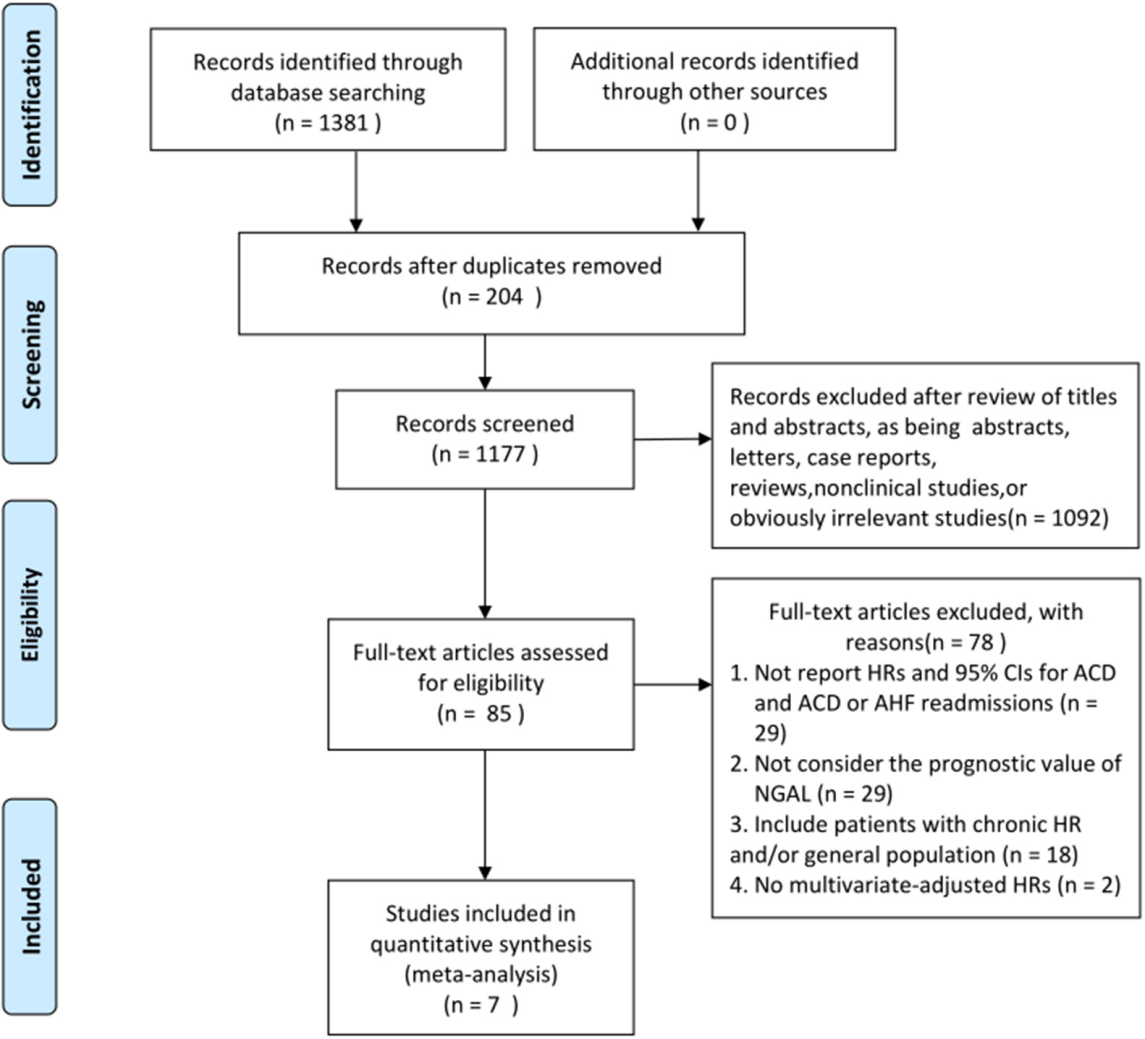
**Flowchart of study selection**. ACD, all-cause death; CIs, confidence intervals; HRs, hazard ratios; AHF, acute heart failure; NGAL, neutrophil gelatinase-associated lipocalin.

**Fig. 2.**
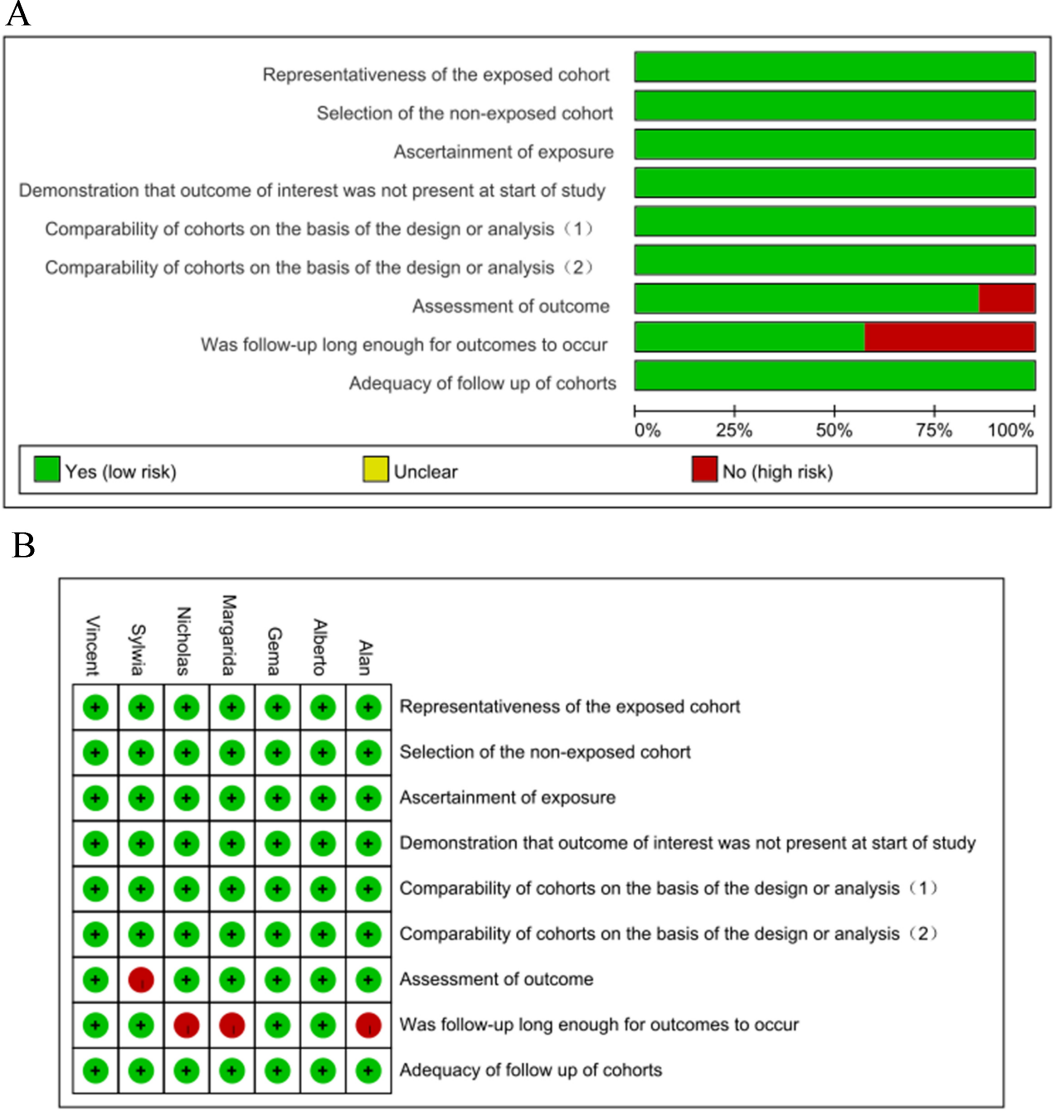
**Quality evaluation of the eligible studies**. (A) Reviewer authors’ judgments are presented as percentages based on the included studies. (B) Review of the authors’ judgments about each domain in each included study.

A summary of the search process is shown in Fig. 1. Initially, 1381 articles were retrieved from the PubMed and Cochrane Library and Embase databases. Seven studies involving 2428 participants were selected for our meta-analysis after meticulously reviewing the articles [13, 14, 17, 18, 19, 20, 21]. Studies were included without disagreements occurring among reviewers. Table 1 (Ref. [13, 14, 17, 18, 19, 20, 21]) shows the basic characteristics of the included studies. In two studies, only patients with reduced LVEF were enrolled [17, 18], whereas in four studies, preserved LVEF was also included [13, 14, 20, 21]. Four of these studies enrolled >200 patients [18, 19, 20, 21], and three studies enrolled <200 patients [13, 14, 17]. All studies were from Western countries [13, 14, 17, 18, 19, 20, 21]. The mean age varied from 69 to 79 years, and the follow-up period ranged from 1 to 36 months. One study enrolled only patients with normal renal function [21]. The overall mean proportion in all the studies was approximately 60.6% males. Two reported ACD, as well as the composite outcome of ACD or AHF readmissions [13, 19], two reported only the composite outcome of ACD or AHF readmissions [14, 17], and three reported only ACD [18, 20, 21]. Therefore, there were five and four studies for analyses of ACD and the composite outcome of ACD or AHF readmissions, respectively. A summary of the NOS assessments of the eligible studies is shown in Fig. 2.

**Table 1.** **Characteristics of the studies included in the meta-analysis**.

First author	Year	Country	Sample size (n)	Age (years)	Male (%)	HFrEF (%)	LVEF (%)	Follow-up (months)	NGAL assay	NGAL levels (ng/mL)	GFR (mL/min per 1.73 m^2^)	Reported outcomes
Maisel [14]	2011	United States, Netherlands, and Switzerland	186	71 ± 13.8	61	53	NA	1	Alere Inc, San Diego, CA, USA	109 (59–181)	NA	ACD or AHF readmissions
Alvelos [13]	2013	Portugal	120	75.2 ± 12.6	50.8	67.2	35 (25–45)	3	Biosite, Quilaban, Lisboa, Portugal	95 (62–167.5)	40 ± 16.5	ACD; ACD or AHF readmissions
Palazzuoli [17]	2014	Italy	179	79 ± 8	54	100	35 ± 10	6	Alere Inc, San Diego, CA, USA	184 ± 171	NA	ACD or AHF readmissions
van Deursen [18]	2014	Netherlands	562	71 ± 11	61	100	32 ± 14	36	Alere Inc, San Diego, CA, USA	85 (60–123)	54 ± 20	ACD
Miñana [21]	2016	Spain	206	72.3 ± 12	49	46.6	51	36 (12–46)	Alere Inc, San Diego, CA, USA	85 (median)	64.1 (median)	ACD
Wettersten [19]	2020	United States, Netherlands, Switzerland, Greece, Germany, Ukraine, Ireland, Spain, Italy	927	69	62	NA	34 (26–43)	1	Alere Inc, San Diego, CA, USA	135.5 (82–241)	57 (40–78)	ACD; ACD or AHF readmissions
Nawrocka-Millward [20]	2021	Poland	248	70.1 ± 12.6	73.4	64	37 ± 14	12	R&D Systems, Inc., Minneapolis, MN, USA	85 ± 54	NA	ACD

Note: HFrEF, heart failure with reduced ejection fraction; LVEF, left ventricular ejection fraction; NGAL, neutrophil gelatinase-associated lipocalin; NA, not available; ACD, all-cause death; AHF, acute heart failure; GFR, glomerular filtration rate.

### 3.2 NGAL and ACD

**Fig. 3.**
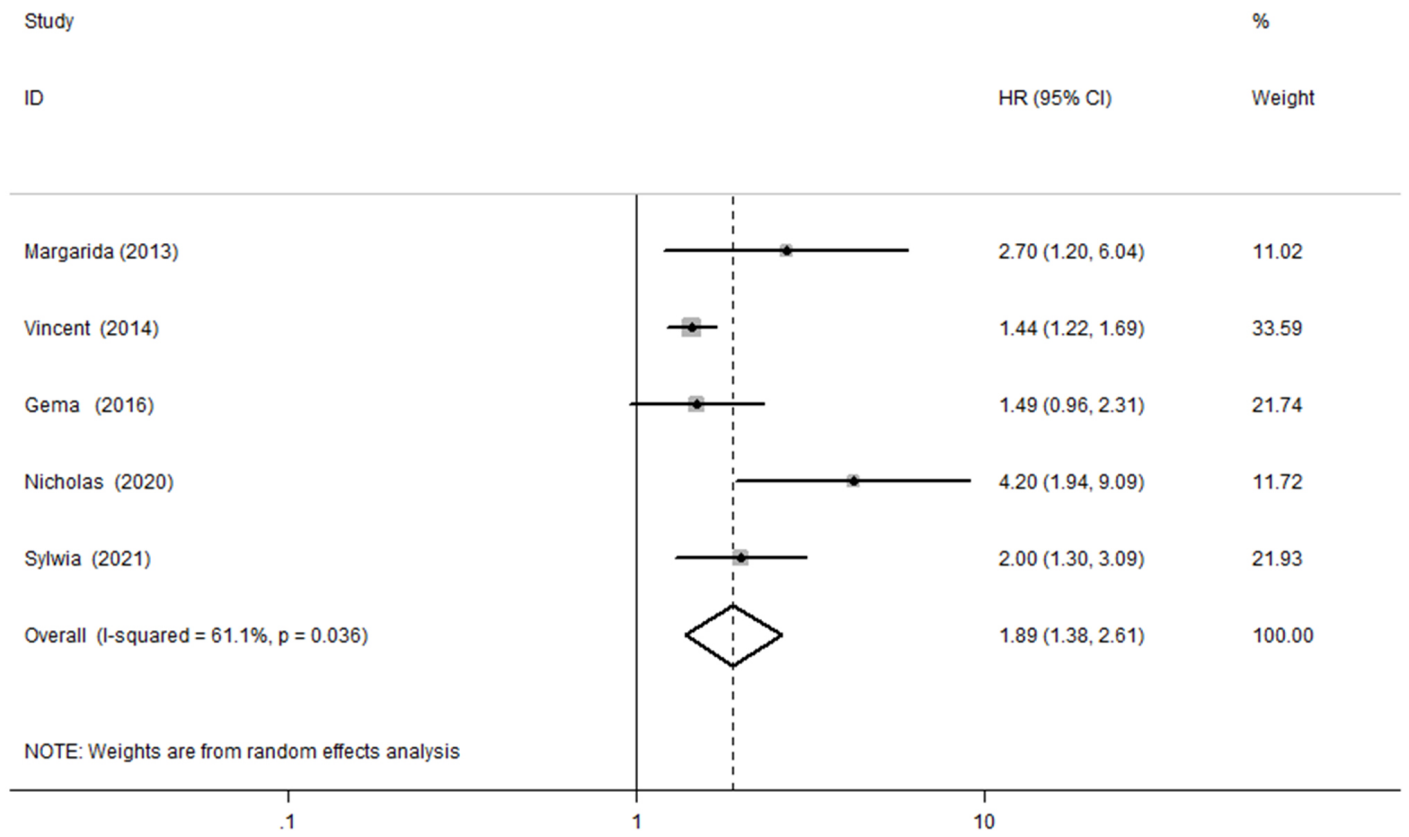
**Meta-analysis of the association between NGAL and the risk of ACD in AHF patients**. NGAL, neutrophil gelatinase-associated lipocalin; ACD, all all-cause death; AHF, acute heart failure; HR, hazard ratio; CI, confidence interval.

Five studies [13, 18, 19, 20, 21] examined the association between NGAL and the risk of ACD in AHF. We adopted a random effects model due to significant heterogeneity (*I*^2^ = 61.1%, *p* < 0.1). Our results showed that patients with AHF with elevated NGAL had a higher risk of ACD (HR, 1.89; 95% CI, 1.38 to 2.61, Fig. 3).

### 3.3 NGAL and the Composite Outcome of ACD or AHF Readmissions

**Fig. 4.**
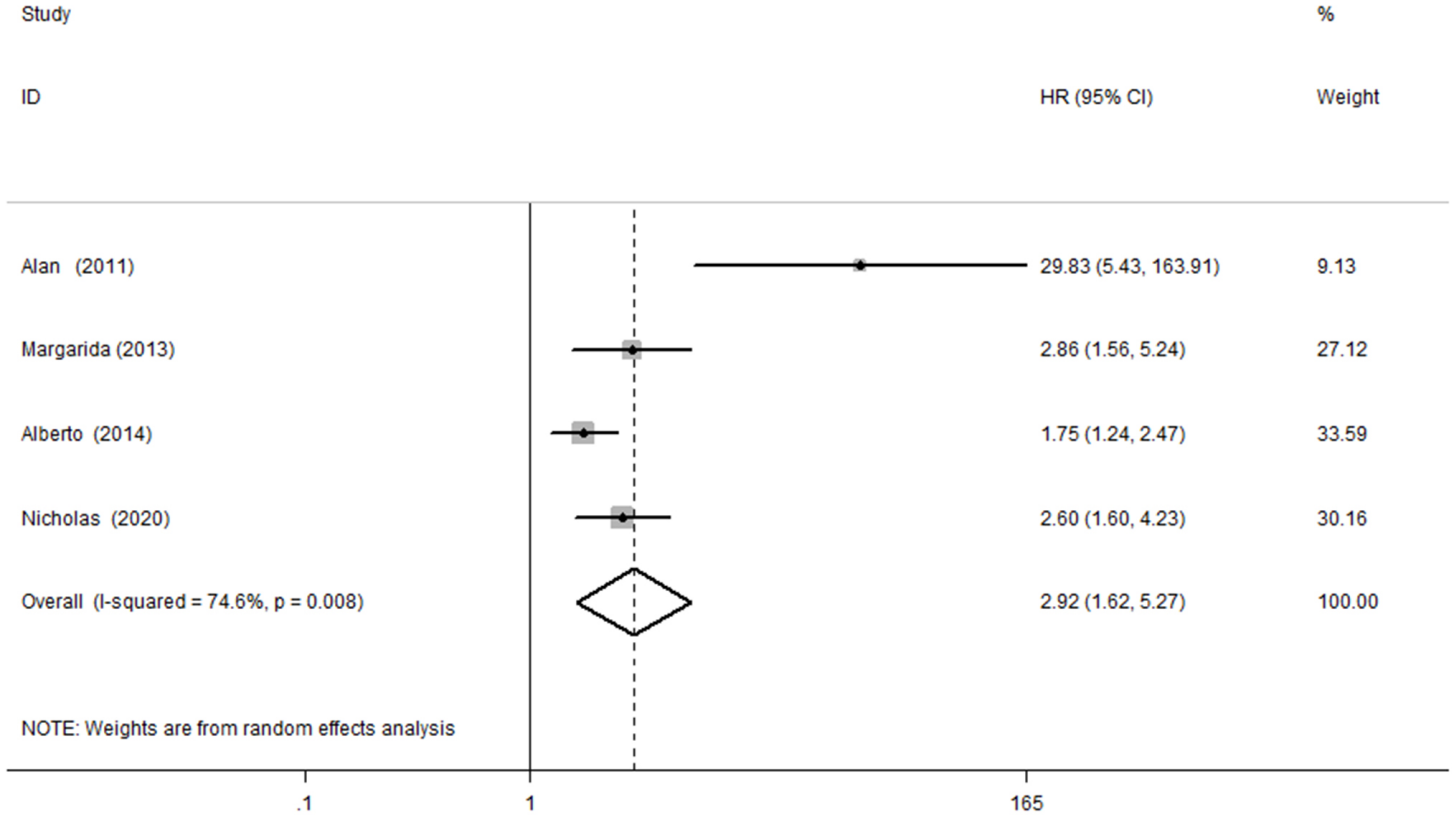
**Meta-analysis of the association between NGAL and ACD or AHF readmission risks in AHF patients**. NGAL, neutrophil gelatinase gelatinase-associated lipocalin; ACD, all all-cause death; AHF, acute heart failure; HR, hazard ratio; CI, confidence interval.

Four studies [13, 14, 17, 19] explored the association between NGAL and the risk of the composite outcome of ACD or AHF readmissions in AHF. Significant heterogeneity was identified (*I*^2^ = 74.6%, *p* < 0.1); thus, we adopted a random effects model. Our results showed that patients with AHF with elevated NGAL were at a higher risk of the composite outcome of ACD or AHF readmissions (HR, 2.92; 95% CI, 1.62 to 5.27, Fig. 4).

### 3.4 Meta-Regression and Sensitivity Analyses

Based on the meta-regression analysis results, no significant association was found between population characteristics (participant age, follow-up time, sample size, cutoff value, eGFR, LVEF, and male sex) and risk for ACD in the five studies that reported the risk (all *p* > 0.05) (**Supplementary Material**).

**Fig. 5.**
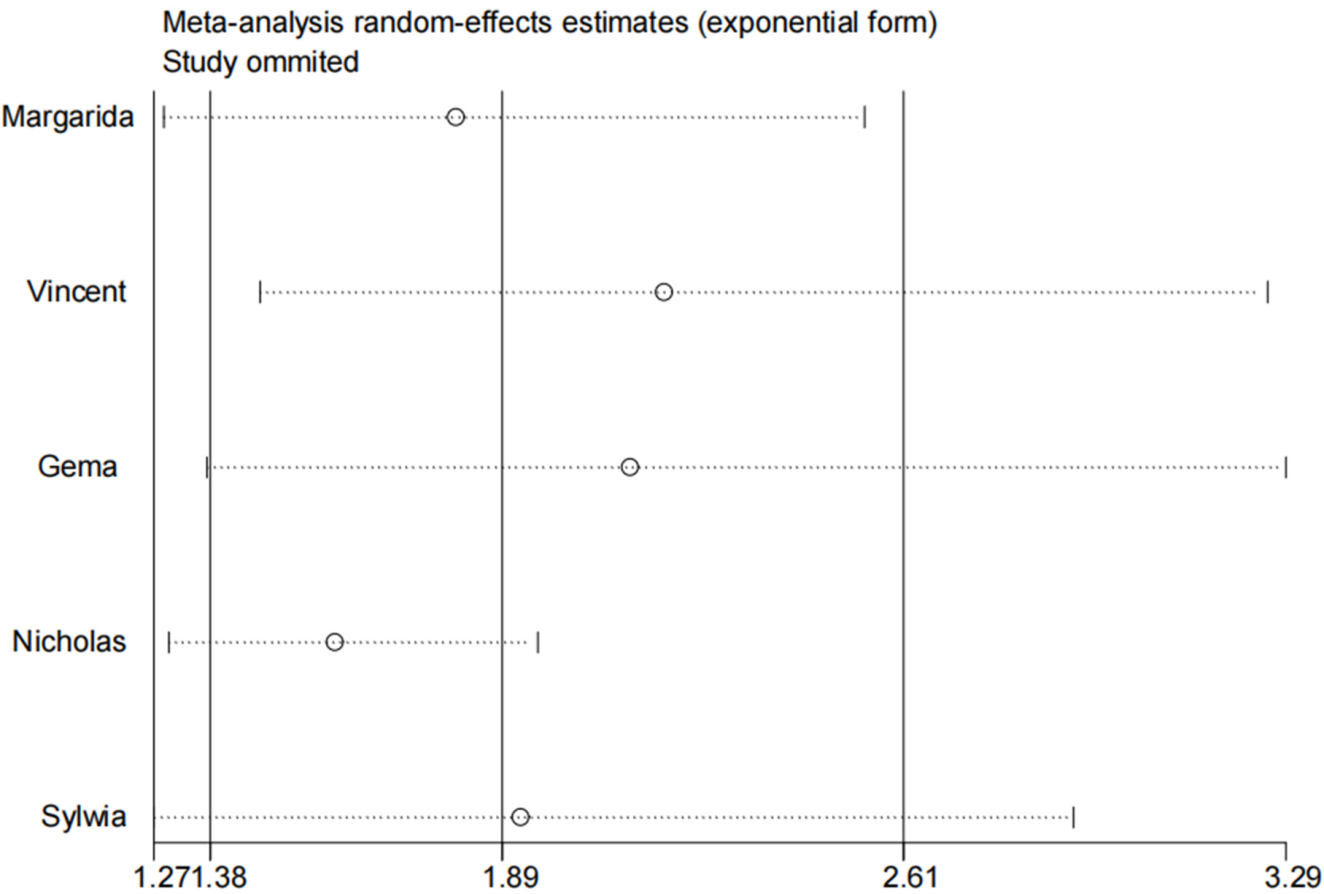
**Sensitivity analysis**.

The sensitivity analysis results confirmed that random or fixed effects models did not affect the association between NGAL and ACD. A sensitivity analysis conducted by omitting one study at a time indicated that the research study by Wettersten *et al*. [19] had the greatest influence on the risk of ACD (Fig. 5).

### 3.5 Analysis of Publication Bias

**Fig. 6.**
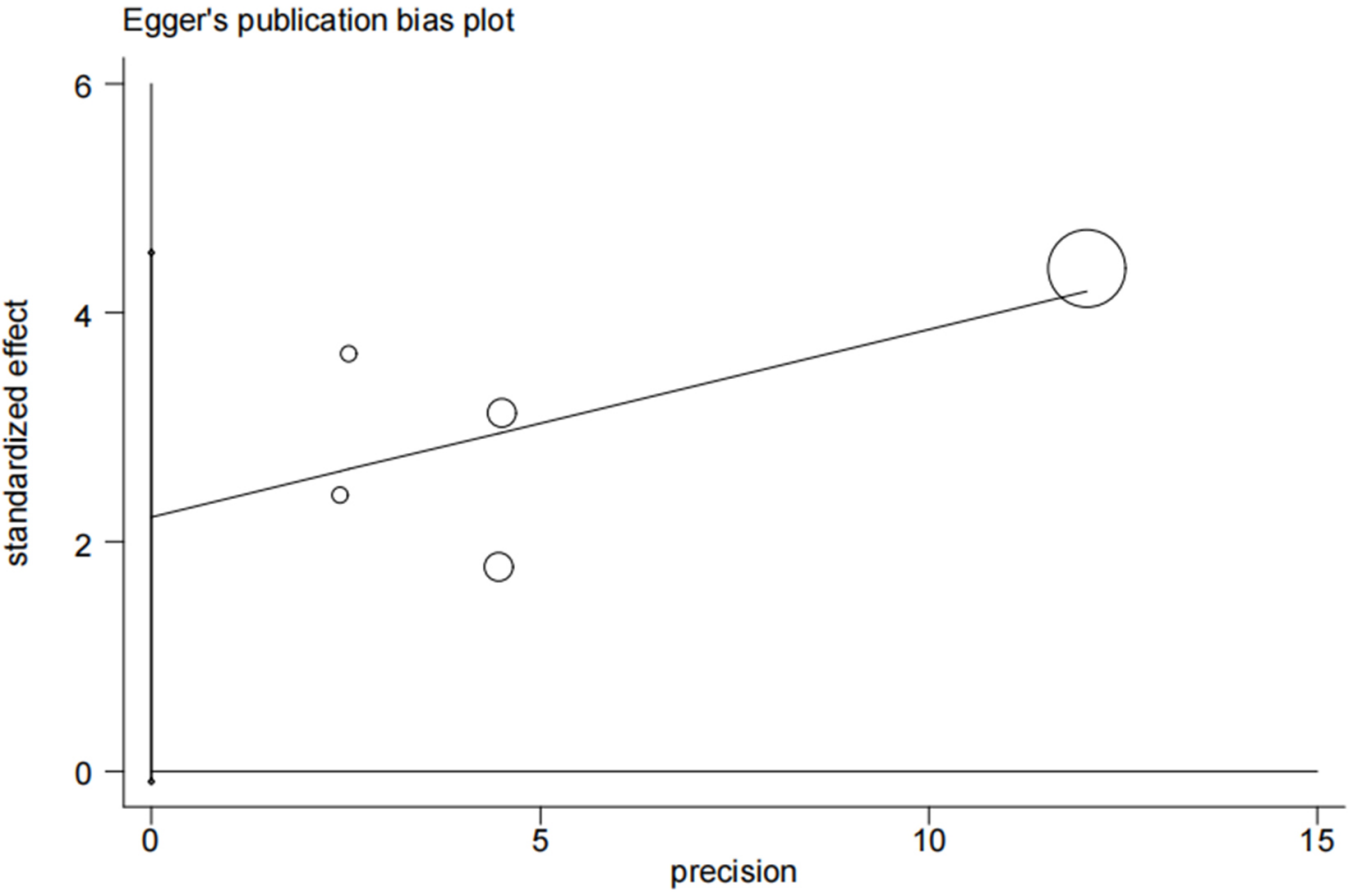
**A funnel plot of the publication bias**.

Publication bias for ACD was not detected when using Begg’s and Egger’s tests (Fig. 6) (*p* >|t| = 0.055 for Egger’s test and Pr >|z| = 0.22 for Begg’s test).

## 4. Discussion

Our meta-analysis combined the results of 2428 patients with AHF from seven studies. According to the aggregated results, serum NGAL levels in patients with AHF are independent predictors of ACD and the composite outcome of ACD or AHF readmissions. The serum NGAL level was associated with an increased risk of ACD in five studies, with a pooled HR of 1.89 (95% CI, 1.38 to 2.61, Fig. 3). In four studies, serum NGAL was associated with the composite outcome of ACD or AHF readmissions, with a pooled HR of 2.92 (95% CI, 1.62 to 5.27, Fig. 4). According to our aggregated results, serum NGAL may be an independent and strong biomarker of AHF prognosis.

In previous studies, serum NGAL was found to have conflicting prognostic properties for short- and long-term outcomes. In a prospective cohort study (GALLANT trial) of 186 patients with AHF, serum NGAL was shown to predict 30-day AHF readmission and mortality independently of serum creatinine and eGFR [14]. Moreover, serum NGAL was an even better prognostic indicator than the brain natriuretic peptide (BNP) [14]. van Deursen *et al*. [18] found that serum NGAL was an independent predictor of an increased risk of long-term (36 months) mortality in patients with AHF, independent of eGFR and cystatin C. Recently, 927 patients with AHF were enrolled in an international, multicenter prospective cohort study. In multivariate models, the highest tertiles of admission serum NGAL were associated with higher incidences of endpoints (death, death, or HF readmission) within 30 days, independent of confounding factors (admission serum creatinine and N-terminal proBNP) [19]. In contrast to these studies, Miñana *et al*. [21] showed that serum NGAL was no longer a significant predictor of ACD after adjusting for N-terminal proBNP and eGFR. The research on NGAL in AHF is still in its early stages; thus, available evidence regarding NGAL as a predictor of outcomes in AHF is limited and remains uncertain. To our knowledge, this meta-analysis is the first to systematically evaluate the prognostic significance of increased serum NGAL levels in patients with AHF. Through comprehensive searches and strict screening, seven studies involving 2428 participants were selected for our meta-analysis. Our pooled results are consistent with most previous clinical studies. A few previous studies have shown opposite results, possibly due to the small sample size. This meta-analysis further confirms that serum NGAL is a significant predictor of ACD and the composite outcome of ACD or AHF readmission in patients with AHF.

As a lipocalin family member, NGAL is produced by neutrophils, kidney epithelial cells, and hepatocytes. Moreover, NGAL levels are significantly elevated following epithelial damage [26, 27, 28, 29]. In the acute phase of toxic and ischemic kidney injury, NGAL is produced by the renal tubular epithelium and released into the urine and blood [30]. However, serum NGAL is not a kidney-specific biomarker. The presence of elevated NGAL levels has been reported in various cardiovascular diseases, such as coronary heart disease, chronic heart failure, and stroke [31, 32, 33, 34]. Additionally, NGAL has demonstrated prognostic value in patients with AHF [13, 14, 17, 18, 19, 20]. Our meta-analysis revealed that higher serum levels of NGAL were associated with higher risks of ACD and the composite outcome of ACD or AHF readmissions in patients with AHF. It is possible to interpret the prognostic value of NGAL as indicative of impaired kidney function. Furthermore, NGAL is also produced in other organs or tissues during systemic inflammation, such as the lung, intestines, skin, failing myocardium, and atherosclerotic plaques [35], which may increase the incidence of death and adverse events. Therefore, serum NGAL should probably be considered an important yet unspecific biomarker of AKI since it also reflects a heightened cardiovascular risk and systemic inflammation. AHF is a clinical syndrome that affects many organs and tissues. Consequently, NGAL may have a strong prognostic value. However, further studies are needed to determine the exact mechanisms underlying the prognostic role of increased NGAL levels.

This is the first meta-analysis to evaluate systematically the prognostic role of serum NGAL levels in AHF. The findings of this meta-analysis have important implications for clinicians and future research strategies. However, we acknowledge some limitations. First, HR for ACD was highly heterogeneous across the included studies, possibly because of differences in follow-up time and participant characteristics. Second, the methods for assaying serum NGAL were not uniform, and the cutoff values varied accordingly. This might have contributed to some bias in the pooled results. Third, because the number of studies included was limited, we did not conduct a subgroup analysis. Thus, further well-designed studies are needed in various regions and populations to confirm our conclusions. Finally, our study was constrained to studies published in the English language only, so publication bias cannot be excluded.

## 5. Conclusions

In conclusion, our meta-analysis proves that a higher level of NGAL is independently associated with poor prognosis in AHF. We anticipate that NGAL will be widely accepted as a prognostic marker. Furthermore, NGAL may provide insight into individualized treatment responses. In the future, more well-designed prospective cohort studies with large sample sizes should be performed to verify the prognostic value of NGAL. Meanwhile, further studies are needed to explore the mechanisms underlying the potential prognostic role of NGAL in AHF.

## Availability of Data and Materials

The data used to support the findings of this study are included within the article.

## Supplementary Material

Supplementary material associated with this article can be found, in the online version, at https://doi.org/10.31083/j.rcm2512428.
